# Gender and Assigned Role Influences Medical Students´ Learning Experience in Interprofessional Team Training Simulations

**DOI:** 10.15694/mep.2017.000028

**Published:** 2017-02-10

**Authors:** Éva Tamás, Samuel Edelbring, Carina Hjelm, Håkan Hult, Oliver Gimm

**Affiliations:** 1 Faculty of Medicine & Health Sciences; 2 Karolinska Institutet

**Keywords:** Simulation-based learning, team training, interprofessional learning, emergency care

## Abstract

This article was migrated. The article was marked as recommended.

The advantages of providing standardized education avoiding exposure of real patients to interventions by novices are appealing both from patient safety and teaching aspects, thus medical simulation has become an integrated part of the healthcare curriculum.

We explored the impact of gender and an acting vs. an observing role in simulation on students’ perceptions of learning outcomes, and of simulation as a learning activity.

A prospective survey for graduating medical students participating in a full day simulated team training session was conducted over three terms. The questionnaire addressed issues related to the session, teamwork and simulation training in general. Participation was voluntary and the study was approved by the regional ethics committee.

The overall response rate was 90.8 %. Authenticity and relevance were considered to be high, though male students scored significantly higher both for authenticity and for relevance. Communication and teamwork were considered to be different, depending on gender and assigned role. Female students and students in an acting role were more ready to discuss knowledge gaps, experienced “good” communication significantly more often, and defined their work as teamwork more frequently. The scenarios were found to be more stimulating and motivating by female students and acting individuals. Self-confidence and self-awareness were declared to be more enhanced for male students and for those who were acting during the simulation sessions. Observers and female students scored significantly lower as regards satisfaction with both the extent of the reflection and the individual feedback.

The perceptions of authenticity and relevance of simulation sessions and students’ readiness to discuss knowledge gaps differed between genders. Furthermore, perceived changes in self-confidence and self-awareness seemed to be different. The observing role implies a different kind of learning process, which is not necessarily inferior to learning by acting.

## Introduction

Medical simulation has become an integrated part of the healthcare curriculum in response to a change in patient care characterized by increasing out-patient treatment and increased focus on patient safety. The advantages of providing standardized education avoiding exposure of real patients to interventions by novices are appealing both from patient safety and teaching aspects ( Kunkler, 2006; Satava, 2009).

Today’s patients have complex healthcare requiering multidisciplinary approach. It is therefore necessary to form interprofessional teams to address these challenging issues regarding their health status ( Lumague et al., 2006). Interprofessional education (IPE) has been defined as occasions when “two or more professions learn from, with and about each other” to improve collaboration and the quality of care ( Zwarenstein, Reeves, & Perrier, 2005). IPE is an approach to prepare healthcare students for future interprofessional teamwork ( Falk, Hult, Hammar, Hopwood, & Dahlgren, 2013; Ostergaard, Ostergaard, & Lippert, 2008). Students who have undergone IPE are more likely to become collaborative interprofessional team members who show respect and positive attitudes towards each other and towards others’ work, thereby improving patient outcomes ( Barker, Bosco, & Oandasan, 2005; Barker & Oandasan, 2005). Interprofessional team training improves technical and non-technical skills, the body of professional knowledge, and attitudes towards patient centred care, and influences the culture of health care performance. Furthermore, interprofessional simulation helps to develop students’ professional identity and their ability to understand other professionals’ roles in the clinical practice ( Bridges, Davidson, Odegard, Maki, & Tomkowiak, 2011; Lumague et al., 2006).

The obvious enacting of learning in simulations is based on experiential acting directly in the scenario during a simulation. However, assigning observer roles to learners is common practice due to the increasing number of students and limited faculty resources. By observing their acting peers the students get an overall view of the team and can contribute their perspective in a debriefing session ( Nystrom, Dahlberg, Hult, & Abrandt Dahlgren, 2016; Reime et al., 2016). However, the characteristics of the observer role are largely unexplored in current simulation research.

### Aims

The present study aimed to further explore medical students’ perceptions of simulated team training regarding (1) the assigned role in simulation, (2) gender differences, and (2) relation of learning objectives.

## Material and Methods

Our simulation centre conducts a full day simulation training program on three days every term for graduating medical and nurse students. There are five simulation sessions each day. At the end of the day, every student has participated in every simulation session. The learning objectives of the simulation are to improve teamwork and communication skills in emergency care situations ( Chakravarthy et al., 2011).

The study was conducted prospectively over a period of three terms, i.e. 1.5 years. All students participating in the simulation training were randomly distributed into groups consisting of four or five medical students and two to six nurse students in their final years, altogether five groups.

The teams were rotating through five simulation sessions. Depending on the session, about half of the medical students acted during each scenario and the other half were assigned observing roles. The students themselves decided how to share these roles. The roles were changed after each session, i.e. students who were acting during one session served as observers during the next session. An instructor was present during the entire session. The sessions followed the structure of briefing-simulation-debriefing. During debriefing, feedback was given to the acting individuals both by the instructor(s) and the observers. Each simulation session lasted an hour. The scenarios focused on emergency care situations (see Appendix).

### Questionnaire

A questionnaire ( Table 1), with five-grade scale answer choices, addressing issues (1) related to the session, (2) teamwork and (3) simulation training in general were distributed to the medical students participating in the team training. The same questionnaire was repeatedly given to every student after every session. In addition to the questions shown in Table 1, the students answered questions on which group they belonged to, which session they were evaluating, whether they were female or male, and whether they were assigned an acting or observing role.

Answering the questionnaires was voluntary. The Regional Ethical Committee approved the study (Dnr. 2012/248-031).

### Statistical analysis

A Mann-Whitney test was used to analyse differences between groups and *p* < 0.05 was considered statistically significant. In addition to single item analyses, four groups were formed by 12 of the questionnaire items. One of these groups (perceived outcome) concerns intended outcomes of the simulation and the others concerned aspects that supposedly influence this outcome. ( Table 1 - colour codes). These groups were statistically analysed regarding their homogeneity by Mokken scale analysis ( Sijtsma, Emons, Bouwmeester, Nyklicek, & Roorda, 2008) ( Sijtsma Molenaar. Sijtsma, 2002). The dependent grouped variables were *Realism*, *Effective team* and *Engagement*, and the outcome variable was *Perceived outcome.* Mokken’s homogeneity criteria of coefficient H > 0.5 was considered to be strongly homogeneous. The correlation between dependent and outcome variables was measured by Spearman’s RHO (r _s_).

## Results

A total of 939 medical students participated in the simulation training sessions and 853 students (396 females, 429 males) returned the questionnaire. Thus, the overall response rate was 90.8 %. Some students chose not to answer all questions (e.g., information on the gender), hence the total does not always reach 100%. Table 1 shows median (25 ^th^ and 75 ^th^ percentile) for the questionnaire answers.

**Table 1.** Questionnaire results (Scale 1 to 5: “do not agree at all” - “completely agree”; Coeff. H: homogeneity scale analysis where the scale is strong when H > 0.5)

	Median	25 ^th^ %	75 ^th^%	Coeff. H
***Scenarios***				
It was easy to get used to the manikin	4	3	4	
The settings were realistic	4	3	5	Realism0.66
The scenario felt realistic	5	4	5	
The simulation as such felt realistic	4	4	5	
The scenario was relevant for my education	5	5	5	
***Teamwork***				
The communication worked well	4	3	4	Effective team0.55
We/they worked as a team	4	4	5	
It was OK to express doubts	4	4	5	
It was OK to discuss knowledge gaps	4	4	5	
The teamwork was full of surprises	3	3	4	
***Simulation Training in General***				
I felt prepared for the simulation	4	3	4	
The purpose of the scenario was clear	5	4	5	
I/they managed to apply practical skills	4	4	5	
I/they managed to integrate theory & practice	4	4	5	
The scenario was stimulating & motivating	5	4	5	Engagement0.66
I want more such exercises	5	5	5	
My self-confidence has increased	4	3	5	Perceived outcome0.54
My self-awareness has increased	4	3	5	
My attitude towards others has changed	3	2	4	
The reflection afterwards was sufficient	4	4	5	
I got sufficient individual feedback	3	3	4	

### Reality and Relevance

In general, both the scenarios of the simulation sessions and the settings were considered to be authentic by the students independently, whether they had an acting or observing role during the simulation ( Table 2). Male students scored significantly higher for the reality of the simulation (p = 0.001) and for the relevance of the scenario (p < 0.001).

**Table 2.** Questionnaire resultsstratified by gender and assigned role in simulation

	Female	Male	Acting	Observer
	Median (25 ^th^ ;75 ^th^%)			
***Scenarios***				
It was easy to get used to the manikin	4 (3;5)	4 (4;5)	4 (4;5)	4 (3;4)
The settings were realistic	4 (3;5)	3 (3;5)	4 (3;5)	4 (3;5)
The scenario felt realistic	5 (4;5)	5 (4;5)	5 (4;5)	5 (4;5)
The simulation as such felt realistic	4 (4;5)	4 (4;5)	4 (4;5)	4 (4;5)
The scenario was relevant for my education	5 (5;5)	5 (5;5)	5 (5;5)	5 (5;5)
***Teamwork***				
The communication worked well	4 (3;4)	4 (3;4)	4 (3;4)	4 (3;4)
We/they worked as a team	4 (4;5)	4 (4;5)	4 (4;5)	4 (4;4)
It was OK to express doubts	4 (4;5)	4 (4;5)	4 (4;5)	4 (4;5)
It was OK to discuss knowledge gaps	4 (4;5)	4 (4;5)	4 (4;5)	3 (3;4)
The teamwork was full of surprises	3 (3;4)	3 (3;4)	3 (3;4)	4 (3;4)
***Simulation Training in General***				
I felt prepared for the simulation	4 (3;4)	4 (3;4)	4 (3;4)	5 (4;5)
The purpose of the scenario was clear	5 (4;5)	5 (4;5)	5 (4;5)	4 (4;5)
I/they managed to apply practical skills	4 (4;5)	4 (4;5)	4 (4;5)	4 (4;5)
I/they managed to integrate theory & practice	4 (4;5)	4 (4;5)	4 (4;5)	5 (4;5)
The scenario was stimulating & motivating	5 (4;5)	5 (4;5)	5 (4;5)	5 (4;5)
I want more such exercises	5 (5;5)	5 (5;5)	5 (5;5)	5 (5;5)
My self-confidence has increased	4 (3;4)	4 (3;5)	4 (3;5)	3 (3;4)
My self-awareness has increased	4 (3;5)	4 (3;5)	4 (4;5)	4 (3;4)
My attitude towards others has changed	3 (2;4)	3 (3;4)	3 (2;4)	3 (2;4)
The reflection afterwards was sufficient	4 (4;5)	4 (4;5)	4 (4;5)	4 (4;5)
I got sufficient individual feedback	3 (3;4)	3 (3;4)	4 (3;4)	3 (2.5;4)

### Teamwork

Students’ perception of communication and teamwork were different depending on gender and assigned role. Though both male and female students scored relatively high on the five-grade scale, female students were more ready to discuss knowledge gaps (p = 0.001). A difference was revealed in the perception of communication when comparing observing and acting students. Those who had an acting role during the simulation experienced “good” communication significantly more often (p = 0.003) and defined their work as teamwork more frequently (p < 0.001), compared to observers.

It was more acceptable for acting participants to express doubts about their performances (p < 0.001) and they were more open to discussing knowledge gaps (p < 0.001) than observers.

### Simulation training in general

All students, regardless of their gender or assigned role, perceived themselves to have come prepared to the simulation and almost uniformly wanted more simulation training sessions of this kind. The purpose of the scenarios was regarded as clearer by male (p = 0.016) but there was no difference between students having observing or acting roles. The scenarios were found to be more stimulating and motivating by female students (p < 0.001) and acting individuals (p = 0.02). Self-confidence and self-awareness were declared to be more enhanced for male students (p < 0.001, each) and for those who were acting during the simulation sessions (p < 0.001, each).

**Figure 1.**
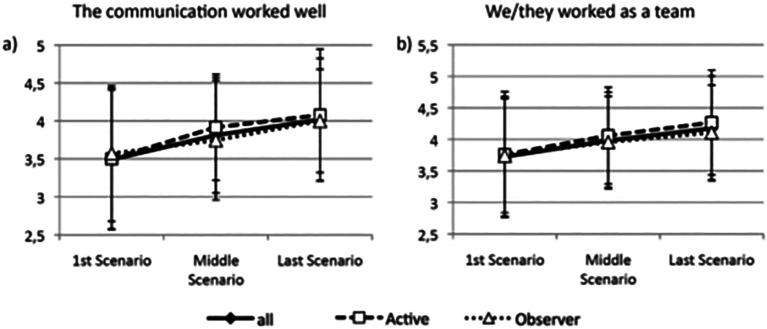
Development of teamwork and communication during the day

While satisfaction with the feedback given to the acting students was scored high (median 4), the individual feedback was scored lower (median 3). Concerning reflection and individual feedback, the students scored highest during the later simulation sessions towards the end of the day ( Figure 1). However, observers and female students scored significantly lower for satisfaction with both the extent of the reflection (p = 0.002, p = 0.04) and the individual feedback (p < 0.001, p = 0.02).

Students in both acting and in observing roles made the assessment that the acting participants were able to apply practical skills to a relatively high extent (median 4) and to integrate theory and practice (median 4) during the scenarios. Nevertheless, female students’ ratings for application of practical skills were significantly higher (p < 0.001) while those for integration of theory and practice were significantly lower (p = 0.014).

### Perceived outcomes

Perceived outcomes (self-confidence, self-awareness, changed attitude) of the learning process were analysed in relation to the grouped variables *Realism, Efficient Team* and *Student Engagement* ( Table 1). The analyses revealed weak correlations between realism, efficient team, student engagement and perceived outcome ( Table 1 & 2).

**Table 3.** Composite variables vs. perceived outcome

Variables	Correlation (r _S_)	p value
Realism - perceived outcomes	0.20	p<0.001
Efficient team - perceived outcomes	0.26	p<0.001
Engagement - perceived outcomes	0.21	p<0.001

## Discussion

### Gender & simulation

Differences in students’ perceptions of communication and teamwork in interprofessional teams relating to gender were revealed and a gender-based difference was also detectable for several aspects of this simulation-based learning activity. Male students assigned higher authenticity and relevance to the simulation sessions than female students did. Lindh Falk et al. studied gender aspects of learning activities at an interprofessional training ward (IPTW) and found that female students, irrespective of their profession, were more positive towards training in an interprofessional environment at a hospital unit ( Lindh Falk, Hammar, & Nystrom, 2015) compared to male students. Our results contradict their findings but can possibly be explained by the different character of the learning activities. Work with real patients at a hospital unit, supported by clinical tutors, can be more appealing for the female student while a simulated environment can be more attractive for male students. Another gender difference concerned the students’ readiness to discuss their knowledge gaps that were revealed during the simulation. Female students turned out to be more open to discussion of these gaps, which proved to be more challenging for men. Nevertheless, bringing these knowledge gaps to light by openly discussing them during debriefing is an important part of the learning process. Furthermore, the female students also encountered application of practical skills to a greater extent than the men but their estimated integration of theory and practice during the session was considerably lower than their male counterparts. It is intriguing that male students to a greater extent felt that both their self-confidence and self-awareness improved as a result of the simulation sessions.

### Assigned role in the simulation

Students having an acting role during the simulation experienced better communication and defined their work as teamwork more frequently compared to observers. It was more acceptable for acting participants to express doubts about the performances and they were more open to discussing knowledge gaps than observers. Studies have reported different learning outcomes between those with an active role and observers. Some investigators have found that the observer role provides the opportunity to practice the reflective clinician role in relation to self-assessment peer reviews and team quality ( Bonnel & Hober, 2016; Hober & University of Kansas). Other reports have shown a lack of student enthusiasm for observing roles ( Harder, Ross, & Paul, 2013). The lack of stress stimulates attention and thus maintains motivation (Hober & University of Kansas.). In addition, studies also exist that have found no differences in the level of knowledge between observers and those students with an acting role ( Jeffries, 2006; Nikendei et al., 2007). A recent study by Reime et al. 2016 showed that participating in different roles is important since the observers reported valuable learning outcomes from watching teams, but on the other hand reported that they would have a better learning outcome from participating and being active in the scenarios ( Reime et al., 2016). The observing students reported that they learned from observations, but also wanted to take an acting role in the simulations to build confidence in their professional roles. Enacting in an observing role in a simulation thus implies a different, but not necessarily less relevant form of learning ( Nystrom et al., 2016). According to Reime (2016), the observers wanted to change roles after the first scenarios and wanted to take an active role. Working in interprofessional teams made the students more prepared to work “in real life”.

### Simulation’s relevance for perceived outcomes

Banerjee et al. 2016 describe successful teamwork implemented in medical school with a simulation program and found that interactive curricular elements improved students’ perception of learning ( Banerjee et al., 2016). They emphasize that this curricular activity is important even for learning about interdisciplinary teams.

All the students were highly enthusiastic and motivated and deemed the simulation sessions realistic. That these factors were found to be associated with perceived outcomes e.g. self-confidence and self-awareness is a relevant finding but probably not very surprising. On the other hand, that working as part of an efficient team has a positive effect on students’ self-confidence, self-awareness and a more favourable attitude for team members of other professions may be a clue for planning simulation-based learning activities in inter-disciplinary settings.

## Conclusion

The perceptions of authenticity and relevance of simulation sessions and students’ readiness to discuss knowledge gaps differ between genders. Furthermore, perceived changes in self-confidence and self-awareness using team training simulations are greater for male students.

The observing role implies a different, but not necessarily inferior kind of learning process than learning by acting.

## Take Home Messages

Gender should be taken into consideration when planning and conducting medical simulation as a learning activity in an interdisciplinary simulation setting.

Being part of an efficient team enhances students’ self-confidence, self-awareness and shift of attitude towards understanding other professions’ roles in the team should be supported. Efforts should be made to establish functional teams to improve learning.

## Notes On Contributors

Éva Tamás MD, PhD is senior university lecturer, vice chair of the executive board for the clinical simulation centre CLINICUM ^®^ at the Faculty of Medicine, Linköping University, Sweden and has a special interest in research questions connected to medical simulation. No conflicts of interest to declare.

Samuel Edelbring PhD is associate senior university lecturer, Director of the clinical simulation centre CLINICUM ^®^ at the Faculty of Medicine and Health Sciences, Linköping University, Sweden. Department of LIME, Karolinska Institutet, Sweden. No conflict of interest to declare.

Carina Hjelm RN, PhD, Department of Medical and Health Sciences, Division of Nursing Science and Department of Cardiothoracic Surgery, Faculty of Medicine, Linköping University, Sweden. No conflicts of interest to declare.

Håkan Hult PhD, associate professor in education, researcher at the clinical simulation center at The Regional Council of Östergötland, and senior researcher at the Department of Clinical science, Intervention and Technology, Karolinska Institutet, Sweden.

Oliver Gimm MD, is a university professor who at the time of the study was a member of the executive board of CLINICUM ^®^ at the Faculty of Medicine, Linköping University, Sweden. He and Håkan Hult initiated and designed this study. No conflicts of interest to declare.

## Appendices

Scenario creation was left to the discretion of the simulation educators leading the sessions.

### Scenario A: Cerebral oedema

Lena (55 years) arrives by ambulance at the emergency room (ER) after she was found unresponsive. She has COPD, Hepatitis C, is a poly drug user. Failing social network. She had a cholecystectomy six weeks before. She was re-hospitalized one week after discharge due to subdural hematoma and pneumothorax after a fall. She was successfully treated and discharged home. After two days at home she was found beside her bed by her neighbour. The paramedics report and leave Lena to the care of the ER nurse. Lena is snoring, unconscious and cyanotic. The nurse immediately alerts her team.

### Scenario B: Hypoglycaemia

An unconscious unidentified malnourished middle-aged woman is brought into the ER of a primary hospital by the ambulance. She was found in bed unconscious by her husband and sent to the hospital. Load and go was performed without further check-up, and the patient was said to “certainly suffer from the usual”. No further information is available.

A-E: The airway is partially obstructed and must be freed by adequate manoeuvres. The patient tolerates an oropharyngeal tube. The respiratory frequency is high and saturation around 75% improving rapidly on oxygen. Heart rate 120/min and BP 80/40 mmHg with a capillary refill of five sec. The patient responds to fluids. Needle marks are found on the patient’s stomach, and multiple haematoma on the patients’ legs as well as general signs of malnutrition and cachexia. Disability is GCS 6 without lateralizing signs, and blood sugar is 0.5 mmol/L. On treatment of this hypoglycaemia, the patient responds with simulated lasting seizures. On therapy with diazepam the patient responds partially, requiring further doses. After 20 mg diazepam, the patient stops fitting but goes into respiratory arrest requiring airway control and ventilation. During the scenario, on consultation (anaesthesia and internal medicine), advice is given by phone but help does not arrive until the patient stops breathing. ECG, CT scans, lab lists and drug lists are provided as asked for in real-time.

### Scenario C: Perforated diverticulitis disguised by cortisone

An almost unconscious but stable female patient has been brought by an ambulance team to the hospital. Her husband is accompanying her. The nurse students can be called any time the medical students wish.

A-E and medical history: tachycardia, hypotension, fever, discomfort in the lower right quadrant of the abdomen. She is suffering from rheumatoid arthritis for which she is taking cortisone but is otherwise healthy. Thus, the patient appears to be in a septic state. With the help of the nurse students, the patient must be stabilized, for example by fluid therapy. Here it is important to brief the nurse students sufficiently since they have not been present from the beginning. If the students do not by themselves exclude common causes of infection as a cause of the septic state (e.g., meningitis, pneumonia), the simulation educator guides them. If the students decide to perform an abdominal CT, they get the information that the patient is suffering from perforated diverticulitis. The misleading findings (lower right abdominal discomfort) and the use of cortisone in this scenario are then discussed.

### Scenario D: Acute coronary syndrome & aortic stenosis

An 80-year-old male with ongoing chest pain, dyspnoea and cough arrives at the ER of a tertiary hospital. He has insulin-treated diabetes mellitus, hypertonia, anaemia and chronic pain in his hips. He is a non-smoker, without risk factors for cardiovascular diseases. On physical examination, he had chest pain, dyspnoea, bilateral crepitation, holo-systolic murmur grade 5/6 in the 2nd right intercostal space and pitting oedema. Diagnostics: ECG showed Left Bundle Branch Block, elevated Troponin-T, chest X-ray as with pulmonary oedema, severe stenosis of the coronary arteries on coronary angiography, severe aortic valve stenosis on echocardiography. Treatment: Adequate painkilling, iv. loop diuretics and nitro-glycerine administration.

### Scenario E: Dissecting ascending aorta

A 60-year-old male who is a heavy smoker with history of myocardial infarction and colitis ulcerosa and depression is found unconscious at home but regained consciousness in a couple of minutes and was transported to the ER of a tertiary hospital within 20 minutes. On physical examination, chest pain, dyspnoea and left hemiparesis are found. Diagnostics: ECG without any sign of myocardial infarction. On ultrasound scan an intimal flap in the right carotid artery and a severe aortic insufficiency with good ventricular function is seen. A CT scan of the thorax shows type-A dissection of the aorta. Treatment: Adequate painkilling, bringing down the blood pressure, securing iv. access. Contact and report to cardiac surgeon.
