# Supplementary material for: Gender and Assigned Role Influences Medical Students´ Learning Experience in Interprofessional Team Training Simulations
Source: MedEdPublish (2016). 2017 Feb 10;6:28. [Version 1] doi: 10.15694/mep.2017.000028 (PMC10885234; doi:10.15694/mep.2017.000028)
Supplement: Peer review history

# Reviewer report: Reviewer response for version 1

**DOI:** 10.21956/mep.19432.r28581

**Published:** 2019-03-30

**Authors:** Ken Masters

**Affiliations:** 1 Sultan Qaboos University

This review has been migrated. The reviewer awarded 3 stars out of 5 A well-constructed study, and a well written paper that explores the perceptions of different genders when medical and nursing students are engaged in IPE in a simulated environment. The authors have laid out the context as described in the literature, and have positioned their research within that context.The value of the research is that it assesses students’ perceptions of the training, and the associations of those data with gender, and it finds important differences. Unfortunately, this paper does not explore whether or not these perceptions reflect the short-term or long-term effectiveness of the training – i.e. whether or not the perceived impact matches the reality of the impact. So, while the students can reflect on their own perception, it is not known whether these differences in perceptions will translate into differences of effectiveness. (Because, if there are no differences, or, if they are reversed, then the importance of these findings are drastically reduced. On the other hand, if the effectiveness of the training matches perceptions, then the perceptions are invaluable as predictors, and can be used to inform remedial action before the students are working directly with patients).As a result, while the paper does have value when dealing with students’ perceptions, I feel that the authors have lost the opportunity to follow through on their work.

Reviewer Expertise:

NA

No decision status is available

# Reviewer report: Reviewer response for version 1

**DOI:** 10.21956/mep.19432.r28580

**Published:** 2017-02-15

**Authors:** Shelley Fielden

**Affiliations:** 1 Leeds Institute of Medical Education

This review has been migrated. The reviewer awarded 3 stars out of 5 This is a well written paper and I found the focus on gender a really interesting one, particularly given the changing gender balance in healthcare professions seen in the UK and elsewhere. This raises some interesting questions for educators in terms of how they develop curricula that acknowledges these differences. It would have been interesting to know the gender difference in the medical and nursing students involved and to have presented the findings by profession and gender. One challenge for those of us developing innovative IPE and researching this area is the assumption that students who have participated in IPE are more likely to be effective patient-centred team members in the future. Logically this may make sense but there is no evidence that this is the case for students involved in IPE at undergraduate level (see 2013 Cochrane review). I would encourage the authors to look outside the medical education literature at sociocultural perspectives on learning (e.g. Lave & Wenger) to enable them to look more critically at IPE in their future work.

Reviewer Expertise:

NA

No decision status is available

# Reviewer report: Reviewer response for version 1

**DOI:** 10.21956/mep.19432.r28582

**Published:** 2017-02-13

**Authors:** John Dent

**Affiliations:** 1 AMEE

This review has been migrated. The reviewer awarded 4 stars out of 5 The substantial study explores the contribution of simulation as a learning activity in interprofessional education and asks whether there is a gender difference in the perceived benefits experienced. Participating students were asked their comments on the exercise in relation to reality and relevance, team working, integration of theory and practice, self-confidence, attitudes to others and opportunity for reflection and feedback.There was a gender difference of opinion on the benefit of being part of an efficient team in relation to enhancing self-confidence, self-awareness and a shift of attitude towards understanding the role of other professions in the team .

Reviewer Expertise:

NA

No decision status is available
